# Notes from the Field: Impact of Increasing the Number of Ebola Surveillance Officers — Kambia District, Sierra Leone, September 2014–September 2015

**DOI:** 10.15585/mmwr.mm6603a7

**Published:** 2017-01-27

**Authors:** Christopher Sandi, Osman Barrie, Hassan Kanu, Foday Sesay

**Affiliations:** 1 District Health Management Team, Ministry of Health and Sanitation, Kambia District, Sierra Leone.

Kambia is one of 14 districts in Sierra Leone. Located in the northwest part of the country, Kambia comprises seven rural chiefdoms. The total population is approximately 344,000. The first case of Ebola virus disease (Ebola) in Kambia occurred on September 4, 2014. Three disease surveillance officers within the District Health Management Team (DHMT) of the Ministry of Health and Sanitation (MoHS) responded to suspected Ebola case alerts. The role of the surveillance officers was to investigate all suspected Ebola cases, and in the event of a confirmed case, initiate the isolation and contact tracing processes. With only one surveillance officer for every 115,000 persons, staffing available for investigation of and response to alerts was inadequate. Without sufficient resources to identify and contain new cases, the Ebola case count continued to increase. To help contain the outbreak, the number of surveillance officers in Kambia was augmented four times. The first addition of a surveillance officer (increasing the number of officers from three to four) occurred in October 2014. By April 2015, the number of surveillance officers had been increased to 25, with two associated rounds of training. MoHS reviewed the number of Ebola cases recorded during September 2014–September 2015 and assessed the impact of the addition of more surveillance officers on the number of Ebola cases recorded in Kambia District.

During September 2014, there were only three surveillance officers in Kambia District, an area approximately 1,200 square miles, and the number of Ebola cases was steadily increasing, with a recorded mean each week of four new confirmed and probable Ebola cases (*1*) (hereafter “confirmed and probable cases” are referred to as “cases”) (Figure). On October 5, 2014, an additional DHMT officer was assigned to surveillance in Kambia, bringing the number of surveillance officers to four; however, the number of cases continued to increase (mean number of cases per week was seven). On November 2, after a total of 48 cases had been recorded, two additional DHMT officers were assigned to surveillance, bringing the total number of surveillance officers in Kambia to six; during the 15 weeks from November 2014 to mid-February 2015, the Ebola epidemic in Kambia reached its peak, with a mean of 10 new cases recorded each week. On February 15, 2015, after 153 additional Ebola cases had been recorded since November 2 (212 cumulative cases), 10 new surveillance officers were trained as chiefdom surveillance officers, permanently based in each of the chiefdoms, bringing the total number of surveillance officers to 16. Three of seven rural chiefdoms were assigned two officers; others were assigned one chiefdom surveillance officer. The goal with adding these 10 surveillance officers was to reduce transportation time between chiefdoms in responding to alerts, and for the officers to act as first responders by ascertaining whether a suspected case met the case definition before the alert was escalated to a headquarters surveillance officer.

**FIGURE:**
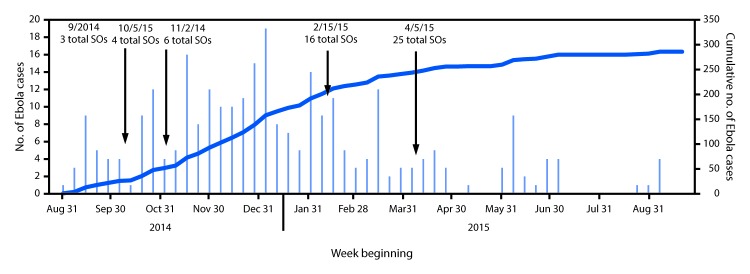
Number of incidents and cumulative Ebola virus disease cases* and the number of additional surveillance officers (SOs) — Kambia District, Sierra Leone, September 2014–August 2015 * Case numbers refer to the sum of confirmed and probable cases.

The number of new Ebola cases in Kambia District began to decline during mid-February–early April, (mean number of cases per week was six). On April 5, 2015, after 244 cumulative Ebola cases had been recorded (almost 2 months since the previous addition of surveillance officers), nine new surveillance officers were trained to operate from headquarters, bringing the total number of surveillance officers in Kambia District to 25. From early April through the first week of September 2015, with 25 surveillance officers in the district (eight times the number at the beginning of the outbreak, and proportional to the number in larger districts, such as Port Loko, which had approximately 40 surveillance officers for a population of 614,000), the number of Ebola cases continued to decline (mean number of cases per week was two). The last Ebola case in Kambia was recorded on September 9, 2015, after a total of 286 cases had been recorded in the district. The two major increases in the number of district surveillance officers coincided with the initial decrease in Ebola cases after the epidemic’s peak, and the second gradual decline to zero cases.

The addition of disease surveillance officers in Kambia enabled public health officials to provide a more timely response to alerts as well as conduct active case searching throughout the district, which was associated with earlier detection and a decline in number of new cases recorded. Active surveillance was combined with outreach and community education from surveillance officers regarding the importance of reporting deaths and raising alerts. The faster response to alerts resulted in early isolation of patients and initiation of quarantine, which limited community spread. Increasing the number of district surveillance officers made early detection and containment possible and led to an eventual end of the Ebola outbreak in Kambia District.
